# Are There Any Relationships Between Abnormal Seminal Parameters and Neutrophil-Lymphocyte Ratio, Platelet-Lymphocyte Ratio, and Red Blood Cell Distribution-Platelet Ratio?

**DOI:** 10.7759/cureus.5242

**Published:** 2019-07-25

**Authors:** Ünal Öztekin, Mehmet Caniklioğlu, Sercan Sarı, Volkan Selmi, Abdullah Gürel, Ayşen Caniklioğlu, Levent Işıkay

**Affiliations:** 1 Urology, Bozok University Faculty of Medicine, Yozgat, TUR; 2 Biochemistry, Bozok University Faculty of Medicine, Yozgat, TUR

**Keywords:** abnormal sperm analysis, male infertility, neutrophil, lymphocyte, red blood cell distribution width

## Abstract

Objective

The aim of this study is to determine the relationship between neutrophil/lymphocyte ratio (NLR), platelet/lymphocyte ratio (PLR), and red blood cell distribution width (RDW)/platelet ratio (RPR) values, which are pro-inflammatory markers, with abnormal sperm parameters, and to evaluate their availability as predictive markers.

Materials and methods

A total of 160 patients, 80 of whom were the control group, formed with match-pair analysis (Group 1), and 80 patients with abnormal sperm analysis, who met the study criteria (Group 2), were included in the study. Complete blood count results were recorded. NLR, PLR, and RPR values were calculated using hematological parameters, and a comparison was made between the two groups.

Results

The mean age was 31.23 ± 5.1 years in Group 1 and 31.33 ± 6.4 years in Group 2. NLR values were 1.84 ± 0.57-1.87 ± 0.65 (P =0.77), PLR values were 105.42 ± 23.89-111.42 ± 34.54 (P = 0.62) and RPR values were 0.05 ± 0.009-0.05 ± 0.01 (P =0.45), respectively. There was no statistically significant difference between the groups.

Conclusions

We investigated whether NLR, PLR, and RPR results can be used as a predictive marker on abnormal sperm parameters. We do not recommend the use of these parameters as a predictive marker.

## Introduction

Infertility is a condition that can cause significant financial costs and emotional stress by affecting one in seven people worldwide. About half of all cases of infertility are caused by male-related factors. It is estimated that it affects about 10%-15% of couples in industrialized countries [1]. Among the causes of male infertility, environmental factors, such as dietary and toxic substances, genetic disorders, infection, and inflammation have been shown [2].

Inflammatory pathologies are known to cause male infertility, and in this process, the production of highly reactive oxygen species (ROS) can disrupt sperm production and structure [3-5]. ROS are theoretically associated with abnormal sperm. In inflammation, ROS production may increase or affect the structure of sperm by suppressing antioxidant mechanisms. Seminal plasma contains enzymatic antioxidants, such as superoxide dismutase, glutathione peroxidase/glutathione reductase system, and catalase, and nonenzymatic antioxidants such as ascorbate (vitamin C), α-tocopherol (vitamin E), taurine, and hypotaurine [6]. During inflammation, these antioxidant mechanisms may cause a condition called ‘oxidative stress,’ which results in sperm damage due to high ROS levels beyond the total available antioxidant capacity in the semen [5].

Oxidative stress has destructive effects on the deoxyribonucleic acid (DNA) of the sperm and membrane, and the inflammatory reactions in the male genital tract can cause male infertility, especially by damaging the DNA of the spermatozoa [2].

Neutrophil/lymphocyte ratio (NLR), platelet/lymphocyte ratio (PLR), and red blood cell distribution width (RDW)/platelet ratio (RPR) are pro-inflammatory markers showing systemic inflammation. It has been shown that NLR has increased in various diseases, and it is widely used to determine the severity of inflammation. PLR is an independent risk factor for reduced survival in various malignancies. RPR has been proposed as a valuable new laboratory test to predict mortality in various diseases [4]. Elevated RDW has been reported to be associated with mortality and other serious adverse outcomes in cardiac, renal, and infectious diseases, even in the general population [7-9].

The study of inflammatory cytokines, such as interleukins, tumor necrosis factor-alpha, interferon-gamma, in seminal plasma may provide more information in understanding the relationship between inflammation and infertility, but the technical difficulties in performing these tests and the cost limit their use in routine practice. The role of NLR, PLR, and RPR in inflammatory diseases has been reported in studies, but the association with abnormal seminal parameters has not been clearly revealed.

The aim of this study is to determine the relationship between NLR, PLR, and RPR values, which are pro-inflammatory markers, with normal and abnormal sperm parameters, and to evaluate their availability as predictive markers.

## Materials and methods

The spermiogram results and inflammatory parameters of patients who were admitted to the urology outpatient clinic for infertility investigation or check-up, and followed up due to the spouse factor infertility between 2017 and December 2018, were also evaluated retrospectively and recorded. After the approval of the local ethics committee, written approval was obtained from all patients and evaluated retrospectively in accordance with the Helsinki Declaration (2017-KAEK-189_2019.01.02_13). The ones who have disorders affecting seminal and/or systemic inflammatory parameters were excluded. Patients younger than 18, with active or chronic inflammatory disease, white blood cell (WBC) > 10000uL, endocrine pathology, undescended testis, karyotype anomaly, ductus agenesis, who have had lateral or bilateral orchiectomy, chemotherapy and radiotherapy history, hematological disease and steroid therapy, have received antioxidant therapy during the six-month period prior to admission, with Grade 3 varicoceles (primary or recurrence) and a history of varicocelectomy in less than one year were excluded from the study. The basic characteristics of patients, age, body mass index (BMI), operation history, previous infertility treatments (antioxidant treatment, intrauterine insemination, in vitro fertilization), drug use history for any reason, and smoking were recorded. Eighty patients, who met the study criteria with abnormal sperm analysis, were included in the study. According to the basic characteristics of the patients, 80 patients with normal spermiogram parameters were involved and match pair analysis was performed. The patients were divided into two groups as the Group 1 (normal spermiogram) and Group 2 (abnormal spermiogram) by taking the guidelines of the 5th edition as a reference, last published in 2010 by the World Health Organization (WHO) [10].

Semen parameters, abstinence time, volume, concentration, total sperm count, progressive motility, total motility, and morphological normal form rates were compared between the two groups. Complete blood count results were obtained with the hematology analyzer (Sysmex XN-1000; Sysmex, Kobe, Japan). NLR, PLR, and RPR ratios were calculated and compared between the two groups.

All statistical tests were calculated using Statistics Package for Social Sciences version 25 (IBM SPSS®, Chicago, IL). Chi-square test was used for categorical data. For the numerical data, the T-test or Mann-Whitney U test was used for independent groups by considering the distribution of the groups. P<0.05 was considered statistically significant.

## Results

The mean age was 31.23±5.1 in Group 1 and 31.33±6.4 in Group 2, and there was no statistically significant difference between the groups. There were no significant differences between the groups in terms of BMI, operation history (varicocelectomy, which is performed before more than one year, ocular and extremity surgery, and tonsillectomy), previous infertility treatment (antioxidant treatment, intrauterine insemination, in vitro fertilization), history of drug use (anticoagulant drug usage was recorded for only two patients), and smoker according to the basic characteristics of the patients. There were 24 patients who performed varicocelectomy before more than one year and 13 (52%) of them were in Group 2 (Table 1).

**Table 1:** Comparison of demographic data between two groups * BMI: Body Mass Index, † SD: Standard Deviation

	Normal Spermiogram [n=80]	Abnormal Spermiogram [n=80]	P
Age [mean] [±SD] †	31.23±5.1	31.33±6.4	0.71
BMI * [mean] [±SD] Normal Overweighted Obese Overall	22.75±1.68 27.56±1.37 32.86±2.70 26.55±3.86	22.18±1.81 27.38±1.44 33.69±3.20 26.22±4.55	0.81
Operation History [n] [%]	22 [27.5]	21 [26.2]	0.72
Infertility Treatment [n] [%]	9 [11.2]	11 [13.7]	0.81
Drug Usage [n] [%]	0 [0.0]	2 [2.5]	0.36
Smoker [n] [%]	50 [62.5]	47 [58.7]	0.93

The data on the seminal parameters of the patients included in the study are shown in Table 2. There was no statistically significant difference between groups in terms of abstinence time and sperm volume. A statistically significant difference was found among sperm concentration, total sperm count, progressive motility, total motility, and normal sperm morphology (P < 0.05).

**Table 2:** Comparison of semen parameter results between two groups D:Day, Ml: milliliter, SD: standard deviation

	Normal Spermiogram [n=80]	Abnormal Spermiogram [n=80]	P
Abstinence time [d] [mean] [±SD]	3.17±0.59	3.23±0.52	0.21
Volume [mL] [mean] [±SD]	3.31±1.34	3.42±1.52	0.85
Concentration [10^6^/mL] [mean] [±SD]	66.05±29.3	10.9±19.8	0.001
Total sperm count [10^6^] [mean] [±SD]	213.3±117.6	41.7±102.3	0.001
Progressive motility [n,%][mean] [±SD]	32.8±10.4	16.7±11.8	0.001
Total motility [n,%] [mean] [±SD]	52.8±11.3	29.3±16.9	0.001
Normal morphology [n,%] [mean] [±SD]	4.14±0.7	1.22±1.08	0.001

There was no significant difference between the two groups in terms of neutrophil, lymphocyte, platelet, and RDW values. There was no statistically significant difference between the NLR, PLR, and RPR groups (P = 0.77, P = 0.62 and P = 0.45) (Table 3).

**Table 3:** Comparison of hematological parameters and rates between two groups * NLR: Neutrophil/lymphocyte ratio, † PLR: platelet/lymphocyte ratio, ‡ RPR: Red blood cell distribution width/platelet ratio

	Normal Spermiogram [n=80]	Abnormal Spermiogram [n=80]	P
Neutrophil count [n][10^3^/mm3] [mean±SD]	4.47±0.13	4.49±0.14	0.91
Lymphocyte count[n][10^3^/mm^3^] [mean] [±SD]	2.51±0.06	2.52±0.07	0.97
Platelet count [n][10^3^/mm^3^] [mean] [±SD]	255.3±5.04	267.1±6.38	0.12
RDW [n][%][mean] [±SD]	12.7±0.09	12.9±0.15	0.45
NLR [n][mean] [±SD]	1.84±0.57	1.87±0.65	0.77
PLR[n] [mean] [±SD]	105.42±23.89	111.42±34.54	0.62
RPR[n] [mean±SD]	0.05±0.009	0.05±0.01	0.45

## Discussion

Inflammation is known to affect the spermatogenesis stages of infertility adversely. Due to the varying levels of inflammatory markers in unexplained infertile patients, low-grade chronic inflammation is often blamed for the etiopathogenetic mechanisms of infertility [11].

Inflammatory conditions are seen in 15% of male infertility cases [12]. Inflammation is the process of responding to injury and tissue damage. This process brings leukocytes and plasma molecules to the sites of infection. Peroxidase-positive leukocytes are the main source of ROS in semen [6]. Failure to remove the infectious agent leads to chronic inflammation. Tissue repair is a characteristic of chronic inflammation and tissue irritation causes tissue destruction. Inflammatory damage in the male genital tract leads to increased ROS [2]. When the testicular tissues of men with idiopathic infertility were examined, it was found that more than 50% of men had leukocyte infiltration [13].

Previous studies have shown that systemic inflammatory processes are associated with significant changes in seminal plasma. Tumor necrosis factor-alpha (TNF-∝) has shown the association of abnormal sperm morphology with low sperm count and motility of inflammatory processes such as interleukin-6 [14-15].

There are various biochemical and hematological markers used to measure systemic infection. It takes a long time to obtain results from many of these markers; in addition, they are costly and their clinical use is limited. NLR, PLR, and RPR are the most common markers of systemic inflammation that have been frequently used in recent years [4,16-17]. These markers are cheap, practical, and widely used markers, which can be easily calculated by dividing the neutrophil, lymphocyte, platelet, and RDW values obtained from routine complete blood count tests.

NLR is a parameter that provides meaningful information about inflammatory conditions and physiological stress conditions, and it has been frequently investigated in recent years for its effectiveness in the prediction of disease, especially in prognosis [18]. Similarly, there are studies reporting that high PLR is associated with poor prognosis in many physiological stresses, in particular, malignancy and neonatal sepsis [19-20]. RPR has been shown as a valuable new laboratory test to predict mortality in acute pancreatitis, hepatic fibrosis, and cirrhosis [21-22]. It has been reported that RPR may be a useful measure to evaluate the disease activity of systemic lupus erythematosus, which is a chronic inflammatory autoimmune disease [23]. In a study evaluating the NLR, PLR, and RPR values in the diagnosis of premature ovarian, insufficiency of which are idiopathic cases in the etiology, it has been shown that NLR can be used as a diagnostic marker [4].

Yucel et al. have investigated the predictive availability of hematological parameters in terms of sperm retrieval in patients who have undergone testicular sperm extraction (TESE). Three hundred and fifty-two patients with non-obstructive azoospermia have been included in the study. Having compared the sperm and non-sperm groups, they concluded that NLR and PLR results were statistically significantly higher in the non-sperm group. They concluded that NLR can be used as an independent factor for the presence of spermatozoa in TESE [17].

In 106 patients, Aykan et al. compared the NLR and PLR results of two groups with normal and abnormal sperm parameters. In the group with normal sperm parameters, NLR results were 1.84±0.78-1.80±0.75 and PLR results were found as 95.32±35.47- 93.57±28.09, respectively. No statistically significant difference was found between them [24]. In our study, we compared the RPR results in addition to the NLR and PLR parameters. According to normal and abnormal sperm parameters, we found NLR results as 1.84±0.57-1.87±0.65 (P = 0.77), respectively, and PLR results as 105.42±23.89-111.42±34.54 (P = 0.62), respectively, and RPR results as 0.05±0.009-0.05±0.01 (P = 0.45), respectively. There was no statistically significant difference in these three parameters between the groups.

It is difficult to predict the infertility potential based merely on sperm parameters. Especially, inflammatory processes have been blamed for infertility, whose causes are unknown. In our study, we have investigated the relationship between inflammatory markers and patients with abnormal sperm parameters in accordance with the normal group, by using complete blood count parameters, which are simple and inexpensive.

There are some limitations due to the retrospective nature of our study. Firstly, active smokers were recorded, but there are no detailed records of the quantity and duration of smoking. And other systemic inflammatory markers, such as TNF-∝ and interleukins, could not be evaluated due to the retrospective study. Another limitation of this study was that results were obtained from the experience of a single-center institution and recorded.

## Conclusions

We have concluded that NLR, PLR, and RPR results cannot be used as a predictive marker on abnormal sperm parameters. However, prospective randomized controlled studies with different markers and higher patient numbers are required to evaluate and clarify the inflammatory causes that occupy an important place in the etiology.
